# Increased Virulence of Bloodstream Over Peripheral Isolates of *P. aeruginosa* Identified Through Post-transcriptional Regulation of Virulence Factors

**DOI:** 10.3389/fcimb.2018.00357

**Published:** 2018-10-26

**Authors:** Caitríona Hickey, Bettina Schaible, Scott Nguyen, Daniel Hurley, Shabarinath Srikumar, Séamus Fanning, Eric Brown, Bianca Crifo, David Matallanas, Siobhán McClean, Cormac T. Taylor, Kirsten Schaffer

**Affiliations:** ^1^Conway Institute, University College Dublin, Dublin, Ireland; ^2^School of Public Health, Physiotherapy & Sports Science, University College Dublin, Dublin, Ireland; ^3^Systems Biology Ireland, University College Dublin, Dublin, Ireland; ^4^School of Medicine, University College Dublin, Dublin, Ireland; ^5^Biomedical and Biomolecular Science, University College Dublin, Dublin, Ireland; ^6^Department of Clinical Microbiology, St. Vincent's University Hospital, Dublin, Ireland

**Keywords:** infection, virulence, bloodstream, pseudomonas, proteomics

## Abstract

The factors influencing the virulence of *P. aeruginosa* in the development of invasive infection remain poorly understood. Here, we investigated the role of the host microenvironment in shaping pathogen virulence and investigated the mechanisms involved. Comparing seven paired genetically indistinguishable clinical bloodstream and peripheral isolates of *P. aeruginosa*, we demonstrate that isolates derived from bloodstream infections are more virulent than their peripheral counterparts (*p* = 0.025). Bloodstream and peripheral isolates elicited similar NF-kB responses in a THP-1 monocyte NF-kappaB reporter cell line implicating similar immunogenicity. Proteomic analysis by mass spectrometry identified multiple virulence and virulence-related factors including LecA and RpoN in significantly greater abundance in the bacterial supernatant from the bloodstream isolate in comparison to that from the corresponding peripheral isolate. Investigation by qPCR revealed that control of expression of these virulence factors was not due to altered levels of transcription. Based on these data, we hypothesize a post-transcriptional mechanism of virulence regulation in *P. aeruginosa* bloodstream infections influenced by surrounding microenvironmental conditions.

## Introduction

*P. aeruginosa* is an important opportunistic pathogen and the predominant cause of infection in cystic fibrosis (CF) patients. It is a leading cause of hospital acquired infections such as ventilator associated pneumonia, urinary tract, and burn wound infections, with immunocompromised and critically ill patients being most at risk (Vincent et al., 1995; Cullen and McClean, 2015). Bloodstream infections (BSIs) are of particular clinical significance due to the high attributable mortality rate which has been reported to range from 26% for community-acquired, to 39% for health care-associated infections (Wisplinghoff et al., 2004; Hattemer et al., 2013). Moreover, BSIs caused by antimicrobial resistant strains have been associated with an increased risk of death (Zhang et al., 2016) and are often associated with a delay in the administration of effective antimicrobial therapy.

Much of the success of *P. aeruginosa* as a pathogen lies in its highly versatile nature, one that can successfully sense and adapt to its surrounding environment. Sites of chronic infection such as the respiratory tract in CF and chronic ulcerative wounds feature variable oxygen gradients and distinct areas of microenvironmental hypoxia to which *P. aeruginosa* has been shown to respond (Worlitzsch et al., 2002). Recent work has demonstrated an alteration in the expression of multiple virulence factors including the potent protein synthesis inhibitor exotoxin A and proteins involved in iron acquisition, on culture of *P. aeruginosa* in hypoxia (Schaible et al., 2017). In addition, a recent study demonstrated a decrease in the adherence to and invasion of epithelial cells by *P. aeruginosa* in hypoxic conditions (Gil-Marqués et al., 2018). As a further example of the influence of environmental features on pathogen virulence, *P. aeruginosa* has been shown to react to physiological levels of the serum protein albumin which is found in significant amounts in acute wound and bloodstream infection sites. The presence of albumin results in a decrease in the expression of *Las*-regulated quorum sensing genes which in turn leads to a reduction in the production of associated virulence factors. Sequestration of secreted homoserine lactone quorum sensing molecules by albumin in the extracellular space is believed to be the mechanism underlying this process (Smith et al., 2017).

Furthermore, in response to iron availability in the extracellular environment, *P. aeruginosa* has a number of iron uptake systems at its disposal to secure this critical nutrient. Pyoverdine and pyochelin are siderophores which are characterized by high and low affinity iron binding capacity, respectively, while pyoverdine also functions as a signaling molecule linked to the expression of virulence factors exotoxin A (ToxA) and alkaline protease (ArpA) (Meyer et al., 1996; Lamont et al., 2002; Nguyen et al., 2014). Other systems triggered for use depending on the iron status of the infection site include the Feo system which is used when infection occurs in microaerophilic environments, and xenosiderophores scavenged from other bacteria in polymicrobial infections (Minandri et al., 2016). As such, an ever-expanding range of metabolic and host factors at the site of infection have been shown to influence the behavior and expression of virulence factors in *P. aeruginosa* and hence may be important in the development of invasive disease.

The development of invasive *P. aeruginosa* bloodstream infection is a serious event and one often seen to arise in the context of a preceding peripheral site infection. Such preceding infections include respiratory and urinary tract, wound, and intravascular catheter associated infections. *In- vitro* studies examining the mechanisms underlying invasion of the bloodstream by *P. aeruginosa* have described the role virulence factors play in aiding the transmigration of bacteria through epithelial and endothelial cell junctions (Golovkine et al., 2017). The type III secretion system (T3SS) effectors ExoS and ExoY have been shown to institute areas of cell death leading to defects in the epithelial cell barrier (Golovkine et al., 2016). The secreted metalloprotease LasB (elastase) cleaves endothelial cadherin (VE-cadherin) and extracellular matrix components thereby disrupting vascular endothelial junctions (Golovkine et al., 2014). These processes together facilitate the passage of *P. aeruginosa* from the initial infection site into the bloodstream. Additional virulence determinants, including cytotoxins, haemolysins, and phospholipids produced by *P. aeruginosa* in response to environmental cues at the infection site, ensure successful immune evasion and progressive invasive infection (Huber et al., 2016; Klockgether and Tümmler, 2017).

In this study, we hypothesized that through microenvironmental influences on virulence factor regulation, isolates from *P. aeruginosa* bloodstream infections may differ in virulence from isolates derived from peripheral sites. To this end, we demonstrated using paired isolates, (a bloodstream and a peripheral isolate both cultured from the same patient) that isolates from the bloodstream microenvironment are more virulent and produce increased amounts of extracellular virulence-related factors in comparison to isolates derived from peripheral site infections. In an era of increasing antimicrobial resistance, bacterial virulence factors shown to influence the course of invasive *P. aeruginosa* disease could be targeted for the development of adjunctive anti-infective agents.

## Materials and methods

### Ethics statement

Ethics approval for the use of bacterial isolates cultured from patient samples, and for the use of the corresponding anonymized clinical information was granted by the Research Ethics Committee of St. Vincent's University Hospital, Dublin, Ireland.

### Bacterial isolates and culture conditions

Clinical isolates of *P. aeruginosa* were obtained from specimens submitted for routine diagnostic purposes to the Department of Clinical Microbiology in St. Vincent's University Hospital (SVUH), Dublin, Ireland, details of which are listed in Table 1. Bacteria were grown statically at 37°C in liquid LB (Luria-Bertani) broth for 48 h following inoculation to 0.1 OD_600_ (optical density measured at 600 nm). The bacterial suspension was centrifuged at 1,700 × g at 4°C for 20 min and the cell- free supernatant generated by passage through 0.22 μM/33 mm sterile filters (Millex Merck Millipore).

**Table 1:** Clinical and microbiological characteristics of seven pairs of *P. aeruginosa* bloodstream and peripheral isolates.

**Pair**	**Type of infection**	**Type of peripheral specimen**	**Hospital or community acquired infection**	**Age sex**	**Pre-existing condition**	**Patient location at time of blood culture**	**AST**	**MLST**
1	Pneumonia	BAL	Hospital	67 M	Lymphoma	ICU	FS	–
2	Peritonitis	Ascitic fluid	Hospital	64 M	Chronic liver disease	ICU	FS	ST-816
3	Pneumonia	Sputum	Hospital	61 M	Leukemia	ICU	MDR	ST-179
4	Catheter related bloodstream infection	Line tip	Hospital	73 F	Metastatic colorectal cancer	Oncology ward	FS	–
5	Biliary sepsis	Abdominal drain fluid	Community	72 F	No	ICU	FS	ST-2720
6	Urosepsis	CSU	Hospital	73 M	HSV encephalitis	ICU	FS	–
7	Catheter related bloodstream infection	Line tip	Hospital	87 F	Lower limb cellulitis	ICU	FS	–

*AST, antimicrobial susceptibility testing; BAL, bronchoalveolar lavage; ICU, intensive care unit; FS, fully susceptible; MDR, multidrug resistant; CSU, catheter specimen of urine; HSV, herpes simplex virus; MLST, multilocus sequence typing; ST, sequence type. –Sequencing studies not performed on these pairs*.

### Virulence analysis using the *in vivo Galleria mellonella* model

The virulence of the cell free supernatant (CFS) was assessed using the sixth (final)-instar larvae of the insect *Galleria mellonella* (greater wax moth) (Livefoods Direct, United Kingdom). Ten larvae per isolate were injected through the last proleg into the haemocoel with 20 μl of CFS. The larvae were incubated at 37°C in the dark for 48 h and death was assessed by lack of movement on stimulation, which was accompanied by complete melanization in most cases. Assessments and recordings of the numbers of live larvae per isolate being tested were made at 16, 20, 24, and 48 h. As a sham control, 10 larvae were injected with sterile LB broth and incubated under the same conditions.

### DNA quantification and quality assessment

Broad quantification and assessment of total gDNA extract quality was assessed using the NanoDrop and 2 μL of extract. Total gDNA from each isolate was serially diluted 1:10 to approximately 19 ng/μL (< 500 ng total in 26 μl) prior to accurate fluorometric quantification using the Qubit and a dsDNA HS (High Sensitivity) assay kit (Thermo Fisher Scientific).

### Whole genome sequencing library preparation

Libraries were prepared using < 500 ng gDNA (19 ng/μL in 26 μL) with the Ultra II FS DNA Library Prep Kit for Illumina (New England Biolabs) and sequenced using the MiSeq platform (Illumina) with a V3 reagent kit (2 × 300 bp paired-end). Library quantity was assessed using the Qubit and fragment length distribution using the 4200 TapeStation (Agilent) and a High Sensitivity D1000 kit (Agilent).

### Bioinformatic analysis

Sequences were trimmed using Trimmomatic 0.38 (Bolger et al., 2014). SPAdes v3.6.2 was used to assemble the trimmed reads for a *de novo* assembly. *In silico* serotyping (Thrane et al., 2016) and multilocus sequencing typing (MLST) were carried out as previously described (Larsen et al., 2012). Trimmed paired reads were mapped to the reference *P. aeruginosa* PAO1 genome by Bowtie2 (Langmead and Salzberg, 2012) Single nucleotide polymorphisms (SNPs) were called by Geneious v8.1.9 (Kearse et al., 2012) with minimum coverage of 5 reads and a stringent minimum variant frequency of 1. Phylogenetic tree construction was performed by Parsnp (Treangen et al., 2014) with the reference genome *P. aeruginosa* PAO1 and using command line parameters -x and -C 5000. Complete and closed *P. aeruginosa* genomes were used in the alignment. Trees were visualized using EvolView v2 (He et al., 2016).

### Mass spectrometry

The samples were prepared and analyzed as previously described (Schaible et al., 2017). Ultracentrifugation of the cell-free supernatant was performed at 100,000 × *g* at 4°C for 70 min (Beckman Coulter) followed by concentration with 3 kDa Amicon ultra filters (Millipore). The concentrate was denatured by adding SDS (sodium dodecyl sulfate) to a concentration of 1%, with 100 mM dithiothreitol, and heating to 95°C for 5 min. Removal of SDS, alkylation and reduction was performed using the FASP (filter aided sample preparation) protocol (Wiśniewski et al., 2009). Peptides were then desalted and analyzed on a Q-Exactive mass spectrometer as previously described (Farrell et al., 2014). The proteins were identified and quantified by MaxLFQ (Cox et al., 2014) by searching with the MaxQuant version 1.5 against the Pseudomonas reference database (Uniprot). Modification included C carbamylation (fixed) and M oxidation (variable). Bioinformatic analysis was performed using Microsoft Excel 2016. Data were obtained from three biological and one technical replicate. Identified proteins were searched against the Pseudomonas Genome Database of PAO1 to identify the corresponding gene, localization within the cell, functional allocations and any known involvement in virulence (Winsor et al., 2016). The mass spectrometry proteomics data have been deposited to the ProteomeXchange Consortium via the PRIDE partner repository.

### Primer design and testing

Primer sequences of 50–150 bp in length were generated using Primer3 web tool (Untergasser et al., 2012) using sequences for target genes (*lecA, rpoN, nrdB, pepP*) obtained from Pseudomonas Genome Database (Winsor et al., 2016). Primers used were as follows: *lecA* forward TGC GCT GGT CAT GAA GAT TG, *lecA* reverse GAA CGA GCC GGA GTT ATT GC; *rpoN* forward CGC GCG ATC ATC AAG AAA CT, *rpoN* reverse CAG GGA TTC GCG GTA TTT GG; *nrdB* forward ACC TGA TCG CCT ACT ACT GC, *nrdB* reverse GCC GCA GTA GAA GAA GAT GC; *pepP* forward GAC ATC ACC CGT ACC TTC CC, *pepP* reverse TTC CAG CAC CAG TTC GTA GA. Primers were synthesized by Eurofins Genomics, Ebersberg and reaction efficiencies tested against *P. aeruginosa* genomic DNA prior to use. To ensure a single product was amplified for all primer sets, PCR products were separated on a 1% agarose gel at 100 V for 40 min. Bands were stained with ethidium bromide and visualized under UV light.

### RNA extraction and quantitative polymerase chain reaction

Following centrifugation of 48-h culture at 1,700 g for 20 min, RNA was extracted from the isolates by resuspension of the bacterial pellet in 1 ml TRIzol® (Invitrogen) followed by separation and precipitation using BCP (1–bromo−3–chloropropane) and isopropanol. The quantity and purity of the extracted RNA was assessed by measuring the absorbance at 230, 260, and 280 nm (Nanodrop, Thermo Scientific). The integrity of the extracted RNA was verified by agarose gel electrophoresis stained with ethidium bromide. Digestion of genomic DNA was performed by use of DNA*-free*™ DNA removal kit (Ambion). cDNA was generated by reverse transcription using random primers. Reverse transcriptase negative controls were also generated. qPCR was run in duplicate on QuantStudio 7 Flex (Applied Biosystems) using the standard cycle for SYBR® green reagents generating standard and melt curves. Bacterial 16S ribosomal RNA (rRNA) was amplified in parallel to correct for differences in the amount of starting RNA.

### Determination of NF-kB activation

THP1-Lucia™ cells which stably express an NF-kB luciferase reporter were treated with the cell-free supernatant of *P. aeruginosa* paired bloodstream and peripheral isolates. Luciferase activity of cells, which corresponds to levels of NF-kB activation, collected at 0, 2, 4, and 8 h, was measured by luminescence using Biolux® Gaussia luciferase Assay kit (New England BioLabs Inc.). Values obtained by luminescence were normalized to the protein absorbance of the cell lysate as measured by BioRad® DC protein assay.

### Statistical analysis

Statistical analysis was carried out using Microsoft Excel 2016 and GraphPad Prism version 5.00 for Windows (GraphPad Software, San Diego). Survival curves were analyzed using the log-rank (Mantel-Cox) test. *T-*tests were used to compare values between paired isolates. Data are presented as mean ± standard error of the mean for at least 3 independent experiments. Statistical significance was denoted as *p* ≤ 0.05.

## Results

### Bloodstream isolates of *P. aeruginosa* are more virulent than peripheral isolates

**Figure 1:**
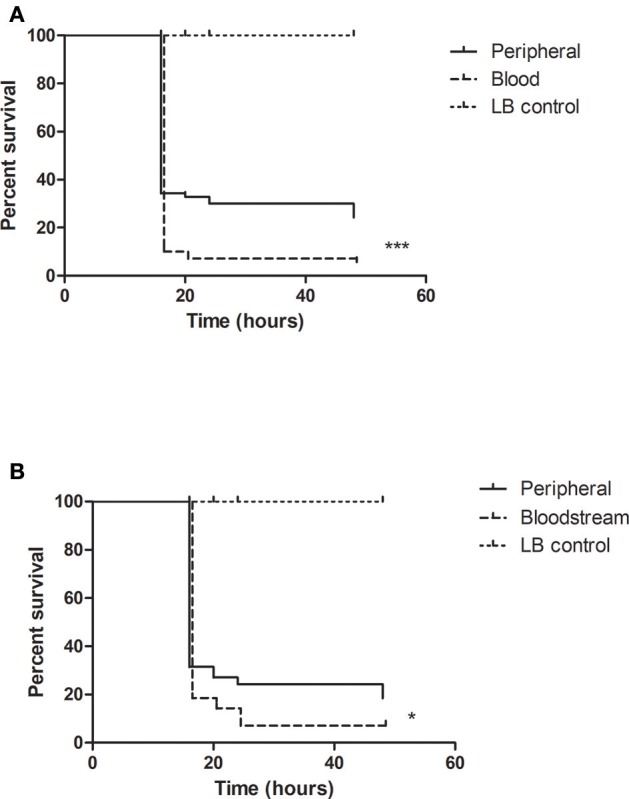
Bloodstream isolates of *P. aeruginosa* are more virulent than peripheral isolates. Survival analysis comparing **(A)** 37 peripheral and 12 bloodstream *P. aeruginosa* isolates, and **(B)** 7 pairs of peripheral and bloodstream *P. aeruginosa* was grown statically in LB broth for 48 h followed by generation of CFS (cell-free supernatant) by centrifugation and sterile filtration of the liquid culture. Twenty microliters of the CFS was injected into the larvae for this experiment. Survival was assessed over a 48-h time period ^***^p < 0.0001, ^*^p = 0.025 log-rank log-rank (Mantel-Cox) test (Graphpad Prism).

Using the *G. mellonella* model, we compared the virulence of clinical *P. aeruginosa* isolates cultured from different sites of infection. Sites such as the urinary and respiratory tract are home to distinct microenvironmental conditions which may influence the expression of certain virulence factors by the infecting pathogen. Retrieved isolates were grown in liquid media for 48 h, centrifuged and sterile filtered to remove the bacterial pellet. The remaining supernatants were then used to assess the virulence of these isolates in the *in vivo* model. We found that the isolates derived from bloodstream infections were more virulent in the larval model in comparison to the isolates from peripheral sites *p* = 0.001 (Figure 1A). The peripheral isolates were cultured from different individual patient specimens and consisted of 11 respiratory tract, 9 urinary tract, and 17 wound isolates. Because this was a heterogenous group representing infection at a number of different sites we proceeded to collect paired isolates. These paired isolates whose genetic identity was subsequently analyzed, consisted of 2 isolates of *P. aeruginosa* from 2 different sites in the one patient—a bloodstream isolate from blood cultures, and a peripheral isolate cultured from a specimen taken from the originating infection site (Table 1). We analyzed the supernatants of these paired isolates in the *G. mellonella* model and found that the supernatants from the bloodstream isolates were more virulent than the corresponding isolates from peripheral sites *p* = 0.025 (Figure 1B).

**Figure 2:**
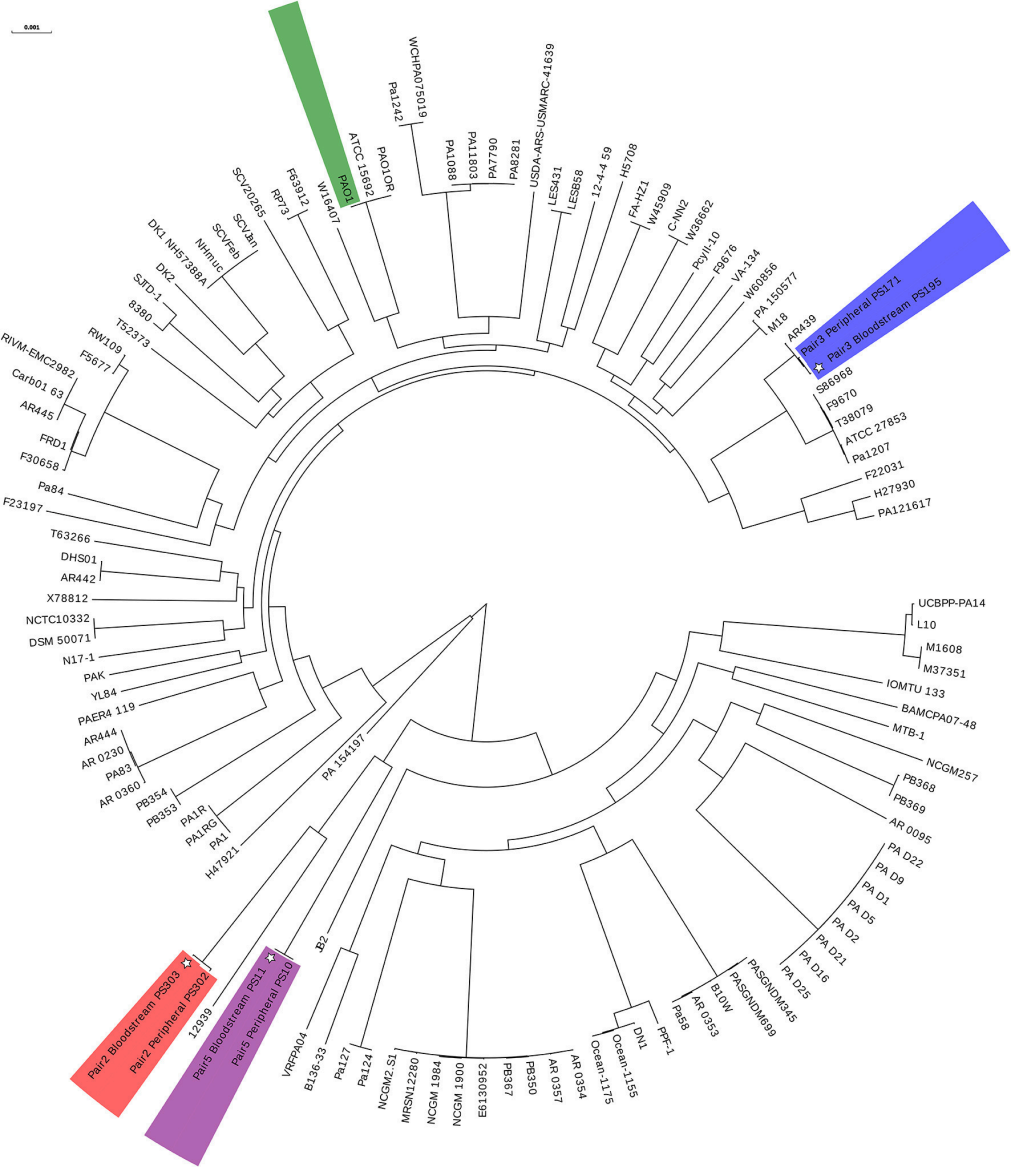
Circular phylogenetic tree showing relatedness of 3 paired bloodstream and peripheral isolates. Whole genome sequencing and phylogenetic analysis performed on 3 out of 7 paired isolates. See Table 1 for further details on paired isolates. Tree constructed using core genome SNPs of 118 *P. aeruginosa* strains. Paired isolates are shown in color with individual laboratory identifiers (PSXXX) listed beside isolate label. PAO1 is shown in green. ^*^bloodstream isolates.

To assess the relatedness of the bloodstream and peripheral isolates within each pair, we performed whole genome sequencing and phylogenetic analysis on three of the seven pairs (Figure 2). This data revealed that each member within the pair shared the same MLST profile and serotype (Table 1) and differed from each other by an average of 5,300 SNPs. In contrast, the difference between these three paired isolates and the reference PAO1 genome was found to be~37,200 SNPs (Supplementary Table 1).

### Bloodstream isolates of *P. aeruginosa* produce increased levels of virulence-related factors

**Figure 3:**
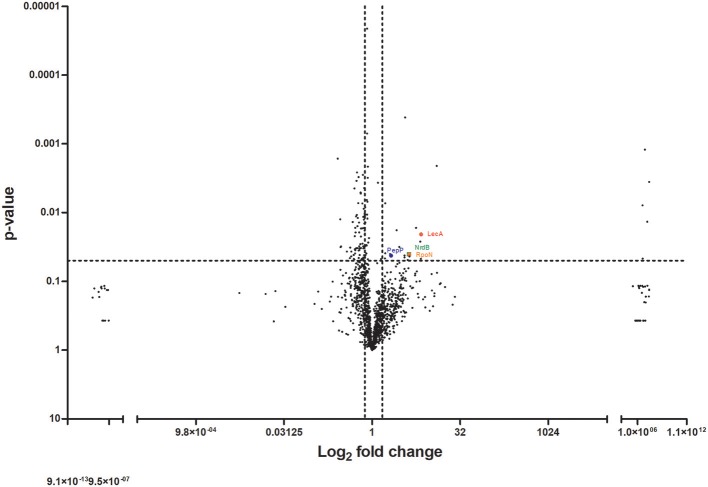
Proteomic analysis of the cell-free supernatant of a pair of peripheral and bloodstream *P. aeruginosa* isolates by mass spectrometry. Volcano plot demonstrating differentially abundant proteins detected and identified from bloodstream and peripheral isolate of Pair 2 (Table 1). Dotted line(s) through y-axis at cut-off for significance at *p* = 0.05, and through x-axis at +/− 1.5-fold change in protein abundance. Proteins identified and quantified following concentration with 3 kDa +/− filters. Significance determined by 1.5-fold difference in abundance with *p* ≤ 0.05 by paired two-tailed *t-*test.

To understand the factors involved in the increased virulence observed with the group of isolates derived from bloodstream infections, we performed mass spectrometric analysis on the cell-free supernatant of a representative pair of bloodstream and peripheral isolates. This pair of isolates, Pair 2 (Table 1), was selected for proteomic analysis as it was the pair that demonstrated the greatest difference in virulence between the bloodstream and peripheral isolate when tested in the *G. mellonella* model. Analysis of these two strains therefore was expected to generate information most relevant to this study in relation to differential virulence factor production between paired isolates. Mass spectrometry on the supernatant of this pair of isolates identified 1,392 proteins from the bloodstream isolate and 1,371 proteins from the peripheral (ascitic fluid) isolate. Proteins were quantified by MaxLFQ (Cox et al., 2014) using the MaxQuant version 1.5 against the Pseudomonas reference proteome database (Uniprot). Cut-off values of a 1.5-fold difference with a *p*-value of < 0.05 were used to determine a significant difference in the abundance of a particular protein between the two groups. Using these parameters, we identified 93 differentially abundant proteins in total, comprising 24 proteins in greater abundance, and 69 proteins in lower abundance in the bloodstream isolate relative to the peripheral isolate, respectively (Figure 3). Identified proteins were further classified according to functional groups as assigned by the Pseudomonas Genome Database (Winsor et al., 2016).

Proteins found in significantly increased abundance in the supernatant of the bloodstream isolate compared to the peripheral isolate were the adhesion factor LecA and the alternative sigma factor RpoN (RNA polymerase sigma factor 54). We also identified 13 proteins with unknown or yet, undetermined functions in this group (Table 2 and Supplementary Table 2). Of note, of the proteins found in significantly lower abundances in the bloodstream isolate supernatant in comparison to the peripheral isolate, 40 out of a total of 69 were categorized as having unknown functions (Supplementary Table 3). RpoN is an alternative sigma factor that regulates transcription of many growth and virulence factors in response to fluctuating environmental conditions, and as such may have a role in invasion of the bloodstream by *P. aeruginosa* (Totten et al., 1990; Potvin et al., 2008). Among the virulence factors known to be promoted by RpoN are the phenazine compound pyocyanin, and the siderophore pyoverdine (Lloyd et al., 2017). Pyocyanin, a well-established virulence factor in *P. aeruginosa*, functions as a redox-active molecule which damages target cells through oxidative stress (Lau et al., 2004). It also acts as a signaling molecule by increasing the infiltration of immune cells to the site of infection, which leads to further inflammation and tissue damage (Hall et al., 2016). Pyoverdine binds to and sequesters extracellular iron in tissues and facilitates its transports into the bacterial cell for use in crucial metabolic activities. In addition, it too functions as a signaling molecule activating the alternative sigma factor PvdS which mediates the expression of multiple virulence genes including those encoding exotoxin A and the proteases PrpL (PvdS-regulated endoprotease; lysyl class) and alkaline protease (Lamont et al., 2002). In light of our discovery of the increased abundance of RpoN in the supernatant of our representative bloodstream isolate, we sought to investigate a link between virulence and pyocyanin and pyoverdine in our isolates. We henceforth measured both pyocyanin and pyoverdine in the supernatants of the 7 paired bloodstream and peripheral isolates but found no significant difference in levels between the two groups (*p* = 0.977; *p* = 0.639) (Supplementary Material and Supplementary Figure 1).

**Table 2:** Proteins found in significantly greater abundance in bloodstream isolate compared to peripheral isolate.

**Protein ID**	**Peptide count**	**Protein name**	**Gene name**	**Functional group**	**Ratio**
Q9I4H5	5	Succinyl-diaminopimelate succinylase	*dapE*	Amino acid biosynthesis and metabolism	1.676
P48247	15	Glutamate-1-semialdehyde 2,1-aminomutase	*hemL*	Biosynthesis of cofactors, prosthetic groups and carriers	1.657
Q9I067	10	FAD-dependent oxidoreductase	*pauB3*	Carbon compound catabolism	2.616
P57109	6	Maleylacetoacetate isomerase	*maiA*	Carbon compound catabolism	4.037
Q9I5E2	4	Carboxyphosphonoenolpyruvate phosphonomutase	*prpB*	Fatty acid and phospholipid metabolism	3.577
Q05097	2	^*^LecA	*lecA*	Motility & Attachment	6.844
Q9HUV8	13	Phosphoribosylamine–glycine ligase	*purD*	Nucleotide biosynthesis and metabolism	2.024
Q9I4I2	2	^*^NrdB, tyrosyl radical-harboring component of class Ia ribonucleotide reductase	*nrdB*	Nucleotide biosynthesis and metabolism	4.238
P49988	2	^*^RNA polymerase sigma-54 factor	*rpoN*	Transcriptional regulators	4.302
Q9HTW6	16	^*^Aminopeptidase P	*pepP*	Translation, post-translational modification, degradation	2.107
Q9HXH8	2	S-adenosylmethionine:trna ribosyltransferase-isomerase	*queA*	Translation, post-translational modification, degradation	2.910
Q9HT52	2	Probable short-chain dehydrogenase	–	Putative enzymes	6.625
		Hypothetical proteins (Lamont et al., 2002)	–	Hypothetical, unclassified, unknown	>1.5

–*Gene name not assigned*,

* *Virulence-related factors chosen for mRNA analysis in all seven paired isolates. See Supplementary Table 2 for details of unannotated proteins*.

### The increased production of virulence-related factors is not transcriptionally regulated

**Figure 4:**
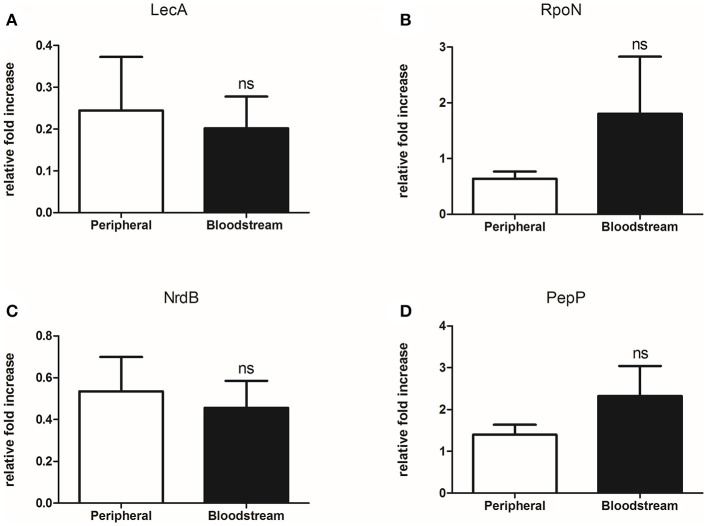
Bloodstream and peripheral isolates produce equivalent levels *of lecA, rpoN, nrdB*, and *pepP* mRNA despite differentially abundant protein levels. **(A)** Comparison of *lecA* mRNA produced by 7 paired bloodstream and peripheral isolates of *P. aeruginosa, p* = 0.780, **(B)** Comparison of *rpoN* mRNA produced by 7 paired bloodstream and peripheral isolates of *P. aeruginosa, p* = 0.281, **(C)** Comparison of *nrdB* mRNA produced by 7 paired bloodstream and peripheral isolates of *P. aeruginosa p* = 0.714, **(D)** Comparison of *pepP* mRNA produced by 7 paired bloodstream and peripheral isolates of *P. aeruginosa, p* = 0.248. qPCR performed using SYBER® green reagents on QuantStudio 7 Flex (Applied Biosystems) using a standard cycle. Primers used designed using Primer3 Webtool (Farrell et al., 2014) with reaction efficiencies tested prior to use. Results are presented as n-fold increase in expression relative to the first sample tested for each gene target. Comparison between the two groups performed using unpaired 2-tailed *t-*test (GraphPad Prism). Data are presented as mean and SEM. ns, not significant.

To assess the level at which the identified differential protein abundance is regulated we selected 4 known virulence factors [LecA, RpoN, PepP (aminopeptidase P), and NrdB (ribonucleoside-diphosphate reductase subunit beta)] for further analysis. The seven paired isolates were cultured in LB broth for 48 h as per the virulence and mass spectrometry experiments, followed by extraction of RNA from the bacterial pellet and subsequent qPCR for the respective target genes. Evidence of *lecA, rpoN, pepP*, and *nrdB* expression was detected in all isolates but no significant difference in mRNA levels was observed when the bloodstream and peripheral groups were compared (Figure 4). As these experiments involved culture of *P. aeruginosa* isolates in artificial media and were not conducted under conditions set to replicate those found in human tissue, we cannot say whether the gene expression profiles observed here reflect those occurring during infection *in vivo*. However, in an in-vitro setting, our findings suggest that increased expression of the 4 virulence-related factors studied (LecA, RpoN, PepP, NrdB) may be regulated at a post-transcriptional level.

### Bloodstream and peripheral isolates activate the innate immune system to equivalent degrees

**Figure 5:**
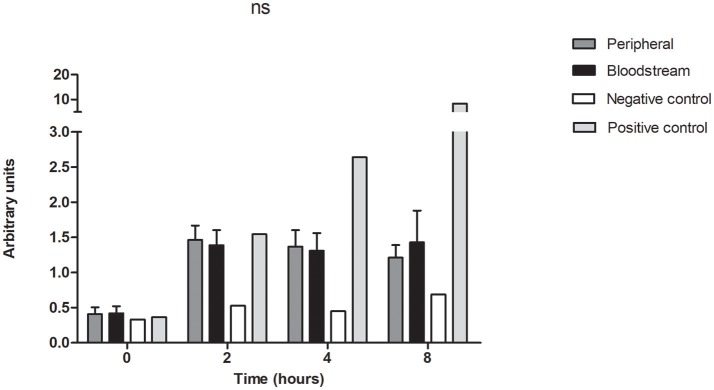
Bloodstream and peripheral isolates produce equivalent activation of NF-kB pathway in human monocytes. THP-1 Lucia® NF-kB cells were treated with the cell-free supernatant of 7 pairs of peripheral and bloodstream isolates. Cells were collected at baseline, 2, 4, and 8 h for quantification of luciferase activity by of Biolux® Gaussia luciferase Assay kit. Values obtained by luminescence were normalized to the protein absorbance of the cell lysate as measured by BioRad® DC protein assay. ns, not significant. Negative control = LB broth, positive control = Pam3CSK4 1μg/ml. Data presented represent mean and SEM of 3 independent experiments.

To assess for a differential host immune response between the isolates cultured from the bloodstream in comparison to the peripheral counterparts in the paired isolates, we measured activation of the NF-kB pathway in a THP1 reporter cell line treated with 20 μl of the *P. aeruginosa* cell-free supernatant. Preparation of the supernatant for this experiment was carried out in the same manner as for the virulence experiments which consisted of culture in LB broth for 48 h followed by centrifugation and sterile filtration of the supernatant. Correlates of NF-kB activation in the THP-1 monocyte NF-kappaB reporter cell line treated with the bacterial supernatants were obtained at baseline, 2, 4, and 8 h, and were equivalent between the two groups of paired bloodstream and peripheral isolates (Figure 5). This finding demonstrates that isolates derived from bloodstream and peripheral sites do not differ in their activation of this aspect of innate immunity.

## Discussion

The identification of factors contributing to the virulence of *P. aeruginosa* in invasive infections is important if we are to select new druggable targets for the development of novel anti-infective agents. In an era of rising antimicrobial resistance, such insight is urgently needed (WHO, 2014). *P. aeruginosa* is equipped with a multitude of virulence factors, the expression of which affects both the development of infection and the determination of disease outcome (Roy-Burman et al., 2001; Hattemer et al., 2013; Jeong et al., 2014). Such virulence factor production varies between bacterial strains but is also strongly influenced by the microenvironment of the infection niche. In the CF lung, challenging conditions are encountered by potential pathogens however, *P. aeruginosa* can successfully adapt to proliferate and survive at this site (Hogardt and Heesemann, 2010). *In-vitro* work has reported a mechanism by which *P. aeruginosa* responds to hypoxic conditions as seen in chronic infections through a reduction in the expression of the virulence factor exotoxin A and proteins involved in iron acquisition (Schaible et al., 2017).

In our study we found that isolates originating from bloodstream infections were more virulent than those from peripheral sites despite these isolates sharing closely related genetic signatures. This suggests that the bloodstream microenvironment might be a determinant that is associated with an upregulation of pathogen virulence. To understand the mechanisms contributing to this observation we performed mass spectrometric analysis on the bacterial supernatants from one pair of isolates. We found that in the bloodstream isolate the abundance of several proteins was significantly increased including factors such as LecA, RNA polymerase sigma factor 54, and proteins important for cellular metabolism and replication. In addition, when we analyzed corresponding mRNA levels, we found that regulation of the production of these proteins is likely to occur at a post-transcriptional level. Interestingly the NF-kB mediated immune response elicited in a human monocyte cell line did not differ between these two groups. Based on these data we hypothesize that the circulatory microenvironment increases the production of certain virulence and virulence-related factors at a post-transcriptional level and this in turn leads to enhanced virulence of *P. aeruginosa* in bloodstream infections.

The adhesion factor LecA (galactophilic lectin, PA-IL) was found at 6.8-fold higher levels in the supernatant of the bloodstream isolate in comparison to that of the peripheral isolate. LecA facilitates adhesion of *P. aeruginosa* to host cells through binding to oligosaccharides of epithelial and endothelial cell surface glycoproteins (Grishin et al., 2015). Infection with lectin mutant *P. aeruginosa* was associated with reduced overall bacterial burden and systemic dissemination in a murine model of acute lung injury (Chemani et al., 2009). Mechanisms supporting a role for LecA in invasive bloodstream infections involve disruption of tight junction permeability in epithelial cell mono-layers (Laughlin et al., 2000), increased alveolar membrane permeability (Chemani et al., 2009), and internalization into lung epithelial cells through binding to cell surface glycosphingolipid Gb3 (Eierhoff et al., 2014). Given the described role of lectins in *P. aeruginosa* pathogenicity, case reports and small studies have described the use of saccharide solutions which act as lectin ligands, in the treatment of human *P. aeruginosa* infection (Grishin et al., 2015). Our study serves to support the further development of such agents targeting *P. aeruginosa* adhesion to and invasion of host cells and tissues in the treatment of *P. aeruginosa* infections.

RNA polymerase sigma factor 54 (RpoN) was an important protein identified in significantly greater abundance in the bloodstream isolate supernatant in comparison to that from the peripheral isolate. As an alternative sigma factor RpoN is involved in the regulation and redirection of transcription initiation in response to fluctuating environmental cues, and has recently been identified as the alternative sigma factor with the single greatest impact on global gene expression in *P. aeruginosa* (Schulz et al., 2015). Specifically RpoN plays a key role in the regulation of several virulence related traits including flagellar-based motility (Totten et al., 1990), the type VI secretion system (T6SS) (Sana et al., 2013), and pyoverdine and pyocyanin production (Lloyd et al., 2017). *rpoN-*negative variants typically exhibit a loss of acute virulence attributes and as such are frequently associated with chronic *P. aeruginosa* infections in CF (Winstanley et al., 2016). To test for a link between virulence factor production and RpoN we measured levels of pyocyanin and pyoverdine in the supernatants of the paired isolates but did not find significantly differential levels to support this mechanism. However, as RpoN effects the expression of a wide range of genes, it may be operating through alternative mechanisms resulting in the enhanced virulence observed with these isolates.

We identified two proteins involved in nucleotide biosynthesis and metabolism in significantly greater quantities in the bloodstream isolate compared to the peripheral isolate. These proteins were PurD (phosphoribosylamine–glycine ligase) which is involved in the *de novo* biosynthesis of purine nucleotides, and NrdB [tyrosyl radical-harboring component of class Ia ribonucleotide reductase (RNR) enzyme] which catalyzes the reduction of ribonucleotides to their corresponding deoxyribonucleotides (dNTPs). The ability to synthesize *de novo* nucleotides has been shown to be crucial for the growth of several pathogens in blood, in particular the *purD* gene was upregulated upon culture in blood of *Streptococcus pyogenes* (Le Breton et al., 2013)*, Staphylococcus aureus* (Connolly et al., 2017), and vancomycin resistant *Enterococcus faecium* (Zhang et al., 2017). In addition, in a recent constructed genome-scale metabolic networks model (GEM) *purD* was among nine genes of the purine biosynthetic pathway in PA14 found to be essential for both bacterial growth and the production of virulence factors such as quorum sensing molecules and components of the O-antigen (Bartell et al., 2017). In total, of the 12/24 proteins we identified as being significantly upregulated in the bloodstream isolate, 7 were assigned to functional groups related to cell growth and metabolism (Winsor et al., 2016). This highlights the recent insight into the link between factors involved in both growth and metabolism, and acute virulence in the pathogenesis of *P. aeruginosa* infections (Bartell et al., 2017).

NrdB protein forms part of class 1 ribonucleotide reductases (RNR) which are oxygen dependent enzymes used for dNTP generation when infection occurs in aerobic conditions (Torrents, 2014). *P. aeruginosa* is uniquely equipped to adapt to environments with variable oxygen availability in that it has genes encoding 3 types of RNRs. Class I RNRs (*nrdAB*) only operate in oxygen rich environments, class II RNRs (*nrdJab*) are oxygen independent, and class III RNRs (*nrdDG*) function in anaerobic conditions (Sjöberg and Torrents, 2011; Crespo et al., 2016). NdrD as a class III RNR enzyme however, was not amongst the proteins found in significantly greater abundance in the supernatant of the peripheral isolate compared to the supernatant of the isolate from the bloodstream environment as might have been expected. Our finding of significantly increased levels of NrdB from the bloodstream isolate may however reflect the availability of oxygen in the circulatory microenvironment in comparison to the peripherally infected site. Acutely inflamed tissues are recognized as having distinct areas of microenvironmental hypoxia due of the metabolic demands of infiltrating immune cells, proliferating bacteria, and vascular flow abnormalities (Schaffer and Taylor, 2015), therefore, the increased abundance of NrdB protein from the supernatant of our bloodstream isolate relative to the peripheral isolate in this pair may be further evidence of the link between cellular metabolism and virulence in *P. aeruginosa* infections. In relation to the regulation of the aforementioned virulence factors in hypoxic conditions, reports on *lecA* and *rpoN* have been described*. lecA* was among the major virulence-related genes induced in a study examining the transcriptional and translational responses of *P. aeruginosa* grown in spaceflight conditions in which an anaerobic mode of growth is most likely adopted (Crabbé et al., 2011). Mutations in *rpoN* are known to occur in the hypoxic conditions found in the CF lung which result in reduced flagellar-mediated motility and alterations in the production of QS signaling molecules (Hogardt and Heesemann, 2010).

We found two proteins involved in translation and post-translational modification processes in significantly greater abundance in the bloodstream isolate in comparison to the peripheral isolate. These proteins were the metalloprotease PepP (aminopeptidase P) and QueA (S-adenosylmethionine:tRNA ribosyltransferase-isomerase). PepP was identified as a crucial virulence-related factor in *P. aeruginosa* by showing transposon mutants of *pepP* displayed reduced swimming ability and killing in a *C. elegans P. aeruginosa* infection model (Feinbaum et al., 2012). In *E. coli* genes encoding *pepP* are involved in the production of outer membrane vesicles (OMV) which play a role in virulence through the transport of materials such as toxins, enzymes and adhesions directly into the host cell cytoplasm (McBroom et al., 2006). In *P. aeruginosa, the* operation of PepP in this capacity has not yet been studied, but if described as in *E. coli*, could provide a mechanistic link to increased virulence in bloodstream or invasive infections.

Exactly 50% (12/24) of the proteins we found in significantly greater abundance in the supernatant of the bloodstream isolate in comparison to that of the peripheral isolate were uncharacterized proteins with unknown functions. As such they may have unidentified virulence-related roles in the pathogenesis of bloodstream or invasive infections. In order to get as much information as possible on the functions and possible effects of the proteome, the search was completed with a study of the homology of the identified unannotated proteins. To this end we used Phyre2, the free web portal for protein modeling, prediction, and analysis (Kelley et al., 2015). This search reported homologies with proteins in other species with variable levels of confidence (Supplementary Table 2) but few with clear virulence associated roles described. Q9HU45 hypothetical protein however, showed homology with part of the periplasmic domain of a sensor kinase in *B. pertussis* (bvgs). This transferase which is involved in activating the expression of multiple virulence-related genes in *B. pertussis* may through further study be shown to have a similar or related role in *P. aeruginosa* (Moon et al., 2017).

Many of the proteins identified by mass spectrometry in the supernatant generated from both isolates including those discussed (LecA, RpoN, NrdB, PepP) are designated to have cytoplasmic cellular locations and are not characteristically known to be secreted via classical means (Yu et al., 2010). Their identification in the extracellular milieu as represented by the cell-free supernatant therefore requires further consideration. Possible explanations include lysis of the bacterial cell with release of intracellular contents into the supernatant, or alternatively secretion by non-classical means such as in outer membrane vesicles (OMVs). OMVs are released by all species of Gram negative bacteria and function to deliver virulence factors such as adhesins and toxins directly into target host cells (Ellis and Kuehn, 2010; O'Donoghue and Krachler, 2016). For instance the sigma factor RpoN is predicted to be found as a protein-DNA complex in the cytoplasm, however a recent study identified a number of sigma factors secreted in membrane vesicles in *S. aureus* (Askarian et al., 2018). RpoN in *P. aeruginosa* may be secreted in a similar fashion hence explaining its identification in the cell free supernatants of our isolates. In *Burkholderia* spp. cytosolic proteins were shown to produce strong humoral responses in chronically colonized Bcc patients, a discovery that supports exposure of the host to intracellular proteins in the extracellular environment (Shinoy et al., 2013). Other proteins may have more than one localization within the cell, LecA for example can also be found on the cell surface where it is a known adhesin (Grishin et al., 2015).

Sixty-nine proteins were found in greater abundance in the supernatant of the peripheral isolate relative to that of the bloodstream isolate in the pair analyzed (Supplementary Table 3). When grouped according to function, and excluding unannotated proteins (40), the highest numbers identified were involved in amino acid biosynthesis and metabolism (6), translation, post-translational modification, and degradation (6), and carbon compound catabolism (4). Differences in nutrient availability between the peripheral and bloodstream microenvironment may explain the strong representation of proteins operating in metabolic activities in this group. *P. aeruginosa* adapts to the nutrient rich airways of the CF lung through the downregulation of a number of metabolic pathways, and such environmental influences are also associated with a change in virulence factor expression (Folkesson et al., 2012).

We selected four proteins identified in significantly greater abundance in the bloodstream isolate in comparison to the peripheral isolate in one of our pairs to assess the regulatory level of these virulence-related factors (LecA, RpoN, PepP, NrdB). In failing to observe a significant difference in mRNA levels to correspond with the protein levels, we surmised that the aforementioned virulence factors were likely regulated at a post-transcriptional level. Discordance between transcript and protein levels is commonly reported across bacterial, fungal and human studies, with variation in mRNA levels in some instances explaining < 50% of the end protein concentration (Laurent et al., 2010; Vogel and Marcotte, 2012). In *P. aeruginosa* the determining effect of the environment on post-transcriptional regulation and post-translational modification of both virulence and metabolic factors is extensively described (Kwon et al., 2014; Ouidir et al., 2016).

Finally, we showed that there was no difference in activation of the NF-kB pathway in THP-1 NF-kB Lucia® cells when the cells were stimulated with the supernatants derived from our paired bloodstream and peripheral isolates (Figure 5). While saturation of the reporter enzyme could lead to equivocal responses in both groups, our experimental positive control Pam3CSK generated much higher levels of luciferase activity, which argues against overwhelming expression as an explanation for the observed lack of difference. As NF-kB is a master regulator of the inflammatory response, it plays a key role in the host's response to infection. Increased NF-kB activity has been associated with increased survival in animal sepsis models of *P*. *aeruginosa* (Sadikot et al., 2006), however in clinical studies, higher levels are often associated with more severe sepsis and increased mortality rates (Arnalich et al., 2000; Liu and Malik, 2006). In *P. aeruginosa* bloodstream infections (BSIs), the increased expression of virulence factors as demonstrated in this study may serve to increase pathogenicity and may play an important role in the high mortality rate associated with this disease.

The limitations of this study are worth noting and firstly include that the bacterial isolates investigated were cultured for 48 h outside of the host and in artificial media—conditions not designed to replicate those encountered *in vivo*. As a result, the factors contributing to the virulence observed in these strains may differ from those expressed at the infection site in human tissues. However, as all isolates were cultured and analyzed under the same laboratory conditions, our findings of increased virulence and virulence factor production in the bloodstream isolates in comparison to those from peripheral sites remain valid. Furthermore, proteomic analysis by mass spectrometry was performed on only 1 of the 7 pairs of isolates in the study. The pair we selected for this analysis demonstrated the greatest difference in virulence between each member of the pair and therefore was expected to give the most insight into the factors relevant to virulence in bloodstream infections. In addition, whole genome sequencing was performed on 3 out of the total 7 paired isolates in this study. The primary aim of this investigation was to confirm the relatedness of the bloodstream and peripheral isolate within each pair rather than to provide data on mutational changes at a genomic level. However, although beyond the scope of this current study, extending whole genome sequencing analysis to all 7 pairs to identify such virulence-driving mutations would be highly informative.

In summary, we demonstrate that the virulence of *P. aeruginosa* isolates from bloodstream infections is increased relative to their peripheral counterparts and may be associated with the increased production of virulence-related factors including LecA, RpoN, and proteins involved in cellular metabolism and replication that have previously been associated with invasive infections. The work outlined here contributes to the growing understanding surrounding the influence of the infection microenvironment on the virulence of bacterial pathogens and supports further study into factors relevant for the progression to invasive P. aeruginosa disease in order to facilitate the development of novel agents for use against this increasingly drug resistant pathogen.

## Author contributions

CT and KS conceived and designed the research. CH carried out and analyzed the virulence, virulence factor, qPCR, and *in-vitro* cell culture experiments. BS, EB, and BC provided technical support. DM and CH analyzed the MS data. SM helped with the MS preparation and analysis. DH performed the sequencing. SN and SF performed the bioinformatics analysis. CH wrote the first draft of the manuscript. All authors contributed to the final version.

### Conflict of interest statement

The authors declare that the research was conducted in the absence of any commercial or financial relationships that could be construed as a potential conflict of interest.
