# Supplementary material for: Increased Virulence of Bloodstream Over Peripheral Isolates of P. aeruginosa Identified Through Post-transcriptional Regulation of Virulence Factors
Source: Front Cell Infect Microbiol. 2018 Oct 26;8:357. doi: 10.3389/fcimb.2018.00357 (PMC6212473; doi:10.3389/fcimb.2018.00357)
Supplement: Supplementary file 1 [file Data_Sheet_1.docx]

Supplementary Material

Increased virulence of bloodstream over peripheral isolates of *P. aeruginosa* identified through post-transcriptional regulation of virulence factors

**Caitríona Hickey, Bettina Schaible, Scott Nguyen, Daniel Hurley, Shabarinath Srikumar, Séamus Fanning, Eric Brown, Bianca Crifo, David Matallanas, Siobhán McClean, Cormac T. Taylor, *Kirsten Schaffer**

*** Correspondence:** Kirsten Schaffer

Email: [kirsten.e.schaffer@ucd.ie](mailto:kirsten.e.schaffer@ucd.ie)

**Supplementary Table 1: Number of SNPs identified by whole genome sequencing of 3 of the 7 paired isolates. A) Number of SNPs identified between each isolate within the pair, and B) Number of SNPs identified between each isolate and PAO1 reference genome.**

|  |  | **PS302**  **Pair 2P** | **PS303**  **Pair 2B** | **PS171**  **Pair 3P** | **PS195**  **Pair 3B** | **PS10**  **Pair 5P** | **PS11**  **Pair 5B** |
| --- | --- | --- | --- | --- | --- | --- | --- |
| **A** | **SNPs relative to PAO1 reference** | 46603 | 49448 | 22989 | 23229 | 40562 | 40419 |
| **B** | **Unique SNPs compared to other isolate in pair** | 5235 | 8080 | 2455 | 2695 | 7950 | 7807 |

P, peripheral isolate in pair; B, bloodstream isolate in pair.

**
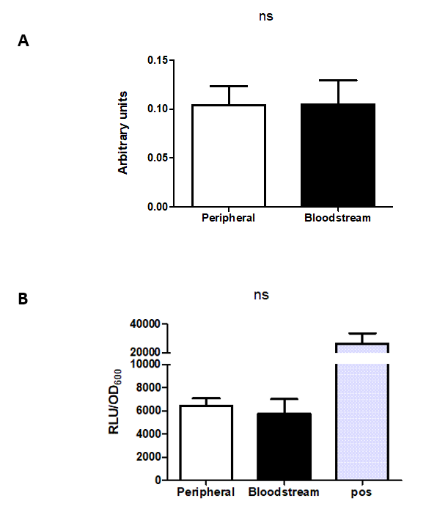
**

**Supplementary Figure 1: Bloodstream and peripheral isolates produce equivalent amounts of A) Pyocyanin, p=0.977 and B) Pyoverdine, p=0.639.** Data showing mean and SEM of 3 independent experiments comparing 7 pairs of isolates. ns= not significant. pos = positive control purified pyoverdine.

**Pyocyanin extraction**: *P. aeruginosa* isolates were cultured for 48-hours in LB broth followed by vigorous shaking and centrifugation at 4000 rpm for 20 minutes and sterile filtration. 2.4 ml of the resulting supernatant were extracted with ml chloroform and subsequently back extracted with 0.8 ml 0.2M HCl. The absorbance of the aqueous phase of the second extraction was measured at 520 nm in duplicate with results expressed relative to the respective OD 600 value of the 48-hour cultures.

**Pyoverdine quantification by fluorescent spectroscopy**: *P. aeruginosa* isolates were cultured for 48-hours in LB broth followed by centrifugation at 4000 rpm for 20 minutes and sterile filtration. 200 uL of cell free supernatant was measured in duplicate in a black 96 well plate (Greiner bio one) using the Spectramax M3 plate reader (Molecular Devices, USA). Levels of pyoverdine were quantified by fluorescence at 425 nm excitation and 530 nm emission and expressed relative to the OD 600 of the 48-hour cultures. Purified pyoverdine (50µM) was used as positive control.

**
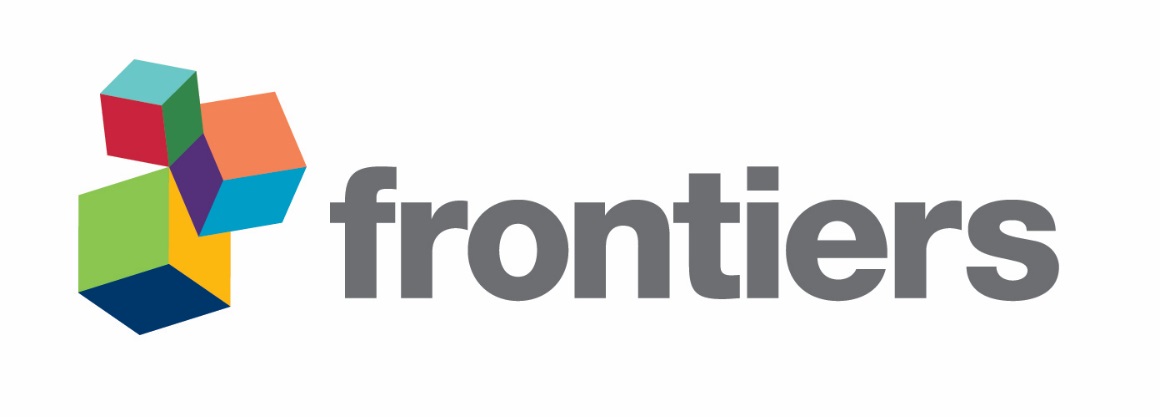
**
